# Contiguity-based sound iconicity: The meaning of words resonates with phonetic properties of their immediate verbal contexts

**DOI:** 10.1371/journal.pone.0216930

**Published:** 2019-05-16

**Authors:** Jan Auracher, Mathias Scharinger, Winfried Menninghaus

**Affiliations:** 1 Department of Language and Literature, Max Planck Institute for Empirical Aesthetics, Frankfurt aM, Germany; 2 Institute for German Linguistics, Philipps-University, Marburg, Germany; 3 Center for Mind, Brain, and Behavior (CMBB), Philipps-University, Marburg, Germany; University of Birmingham, UNITED KINGDOM

## Abstract

We tested the hypothesis that phonosemantic iconicity––i.e., a motivated resonance of sound and meaning––might not only be found on the level of individual words or entire texts, but also in word combinations such that the meaning of a target word is iconically expressed, or highlighted, in the phonetic properties of its immediate verbal context. To this end, we extracted single lines from German poems that all include a word designating high or low dominance, such as *large* or *small*, *strong* or *weak*, etc. Based on insights from previous studies, we expected to find more vowels with a relatively short distance between the first two formants (low formant dispersion) in the immediate context of words expressing high physical or social dominance than in the context of words expressing low dominance. Our findings support this hypothesis, suggesting that neighboring words can form iconic dyads in which the meaning of one word is sound-iconically reflected in the phonetic properties of adjacent words. The construct of a contiguity-based phono-semantic iconicity opens many venues for future research well beyond lines extracted from poems.

## Introduction

While traditional linguistics largely keeps endorsing the hypothesis that the linguistic sign is arbitrary regarding the relation of the signifier (i.e., sound or written characters) and the signified (i.e., meaning) [1], evidence for non-arbitrary sound-meaning relations in the use of language is growing (for reviews see [2–6]). Several studies have tested phonosemantic congruencies between meaning and acoustic characteristics on the level of individual words [7–14] or statistical correlations between the frequency of particular phonemes and the overall emotional tone of entire texts [14–21]. The aim of the present study is to introduce a hitherto unconsidered variant of sound-meaning relations in natural language texts. In this variant, we predict sound-iconic relations between the meaning of a given word and the sound patterns of its immediately neighboring words. That is, we expect that the sound of adjacent words is phonetically an iconic simile for the meaning of a given reference word. We will refer to this kind of phonosemantic relations between adjacent words as *contiguity-based sound iconicity*.

*Sound iconicity*, also known as *phonosemantics*, *sound symbolism*, *linguistic iconism*, or *phonological iconicity*, refers to relations between sound and meaning of linguistic signs (for discussions of the terminology see [22–25]). In this article we will use the term sound iconicity to refer to systematic and universal associations of articulatory-acoustic properties of phonemes with non-acoustic attributes, such as size, shape, or affect. In this meaning, the concept of sound iconicity does not include imitative sound-meaning relations such as in the case of onomatopoeia.

Research on sound iconicity has covered a wide variety of phenomena and approaches, monitoring the relevance of sound iconicity across languages and language families. In experimental approaches, introspective and implicit methods were used to test relations between phonetic properties of pre-lexical stimuli, such as single phonemes or meaningless phoneme combinations and non-acoustic semantic concepts [7, 26–44]. Results provide robust evidence for systematic and language-independent associations between articulatory-acoustic characteristics of phonemes and semantic concepts (but see [45]).

Natural language use has likewise been tested for the relevance of sound iconic associations. Studies on language acquisition suggest that sound iconic words are learned easier and faster in both early first [46–49] and second language acquisition [50–54]. There are also attempts to derive the selection of the best-fitting brand or product names from insights into sound iconicity [55, 56]. Finally, studies on prosody in verbal interactions reported that iconic modulations, such as changes in the pitch of a voice, are not only affected by the emotional state of the speaker or the syntactic structure of an utterance but also by its content [57–59].

Within the research on the relevance of sound-meaning relations in natural language use, a subgroup of studies focused on relations between—mostly emotional—aspects of the content in natural language texts and the relative occurrence of phonemes with specific articulatory-acoustic characteristics. Compared to the studies referred to above, these studies have yielded a less consistent picture. Some of them found a significant relation between the emotional content of texts and their phonetic structure [14, 16–18, 21, 60, 61], others have not [19, 20]. Moreover, the same phonetic characteristics have been attributed to different iconic meanings. For example, a high occurrence of nasal consonants in a text has been linked to “tenderness” in one study [18] and to “melancholy” in another [16].

The present proposal of a novel type of sound-iconic relations in natural language focuses neither on individual words nor on entire texts, but on small clusters of neighboring words. Honoring long-standing assumptions that art shows a particularly strong resonance of form and content [62–69] and specifically following Jakobson’s hypothesis that iconic sound-meaning relations are likely to be more frequently and more saliently found in poetic language [70], we used lines extracted from poems as the first testing ground for our hypothesis. At the same time, and just like Jakobson, we do not imply that poetic language use is categorically set apart from ordinary language use. Rather, we expected that poetry might provide first evidence for a phenomenon that is also relevant in ordinary language use, if only to a lesser degree.

### Sound iconicity of magnitude

In the current study, we tested whether words that refer to either largeness or smallness differ regarding the relative occurrence of specific phonemes in the words immediately preceding and following them. The so-called *sound iconicity of magnitude* is a well-studied phenomenon. Previous studies have provided evidence that high-front vowels (such as /i:/ in ‘heed’) tend to be associated with smallness and low-back vowels (such as /ɑ:/ in ‘bath’) with largeness [26, 35, 38, 40, 71–74]. This cross-modal association between articulatory-acoustic characteristics of vowels and the non-acoustic property of size has been reported for participants of various mother tongues [75, 76] and even for prelingual toddlers [37]. Moreover, comparative studies have found a near-universal tendency for an increased likelihood of high-front vowels in linguistic units that refer to smallness and related concepts [8, 10, 12] (but see [11, 77, 78]; for a discussion see [79]).

**Fig 1:**
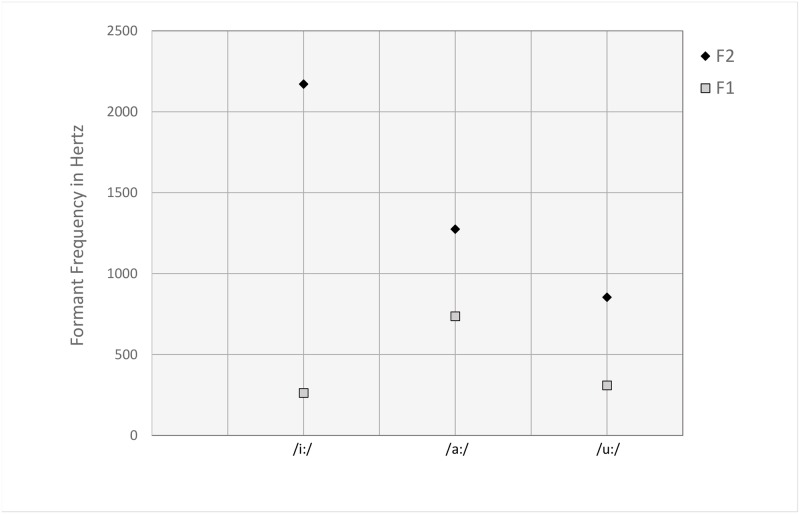
Relation between frequency of the first two formants (F1 and F2) and their articulatory characteristics for three vowels. The distance between F1 and F2 indicates the formant dispersion. As can be seen, the formant dispersion is widest for the high-front vowel /i:/ and considerably narrower for the low vowel /a:/ and the back vowel /u:/.

Sound iconicity of magnitude can also be understood as a specific kind of synaesthetic association, that is, an association across sensory modalities. The characterization of vowels as high vs. low and front vs. back refers to articulatory features related to the relative position of the tongue when pronouncing the respective vowel. These articulatory characteristics are directly related to the frequency of the vowel’s first two formants [80]. Formants are the repercussions of resonance frequencies of the vocal tract and correspond to higher amplitudes in the power spectrum of vowels. The frequency of the formants is influenced by the size and shape of the vocal tract, which, in turn, varies depending on the articulatory movements, such as opening or closing the mouth, changing the position of the tongue, and rounding or spreading the lips. The frequencies of the first two formants and their relative distance are important for characterizing the distinct sound of a vowel. For example, the distance between the first and second formant—the so-called *formant dispersion*—is relatively wide for high-front vowels, such as /i/, and considerably narrower for low vowels, such as /a/, and back vowels, such as /u/ (Fig 1). A closer look at the specific relation between the characteristic frequencies of a vowel’s first two formants suggests that the association between vowels and size corresponds to the relative formant dispersion of the respective vowels: vowels that have a narrow formant dispersion are associated with largeness, whereas vowels that have a wide formant dispersion are related to smallness.

Sound-iconic associations between formant-frequency and size are, therefore, closely related to the *frequency code*, a theory originally introduced by Ohala [81, 82]. Ohala pointed out that, across species and languages, acoustic frequency is used to convey an impression of size. Moreover, as in most species size is directly associated with physical and social dominance, acoustic frequency can also strategically be used to intimidate or appease rival conspecifics or to gain an advantage in the competition for mates (see also [83]). According to Ohala [82], the frequency code is causal for many sound-meaning relations that are seemingly independent of a specific language, culture, or even species, including phonosemantic congruencies in the lexicon, the vocal expression of affect, the use of sound-frequency in threatening vocalization, or emotional facial expressions to display fear or anger. To give an example, facial gestures of primates that express aggression or submission involve lip movements that also affect the frequency of the voice’s formants. For Ohala [82], the original motivation of these gestures is to make the voice sound darker and more intimidating when attacking a conspecific, and brighter and friendly-sounding when trying to appease a dominant opponent (p. 332–333). As the facial gestures described by Ohala are closely related to articulatory movements when pronouncing back or front vowels, the association between formant dispersion of vowels and their association with size, strength, or dominance might well be rooted in facial expressions of emotions.

Formant dispersion, thus, might not be associated with (directly perceivable) size, but more generally with a notion of physical and social dominance. Corroborating this hypothesis, several studies have revealed that body size, via its close relation to the length of the vocal tract, shows a negative correlation with the distance between the frequencies of the formants of an individual’s voice [84]. Accordingly, formant dispersion is widely considered to be indicative of body size across various species [85–91], including humans [92, 93] (but see [94]). A recent overview of studies on various mammal species suggests that males strategically lower their formant dispersion to appear larger and thereby gain an advantage in the competition for mates and access to resources [95]. Similarly, studies with humans have reported that the formant dispersion of the male voice is used to signal physical and social dominance in interactions [96, 97]; and that it predicts females’ preferences regarding male voices [98–101]. Thus, it appears that the naturally occurring relation between formant dispersion and body size initiated the development of use of formant dispersion as a communicative tool to signal physical and social dominance.

There is also evidence that these relations between sound frequency and physical and social dominance may have been adopted in sound-iconic associations with vowels. Fischer-Jørgensen [102], for example, found that the relative formant dispersion of vowels corresponds to how these vowels are assessed on a variety of scales, such as dark–bright, hard–soft, or small–big. In a more recent study [26], participants showed a significant tendency to implicitly associate front vowels with fearful body postures and back vowels with angry and aggressive behavior. As both anger [103] and fear [104, 105] have been suggested to be closely related to a dominance–submissiveness system facilitating the establishment of dominance hierarchies (see also [106–112], these findings again support the assumption that formant dispersion is used to signal physical and social hierarchy.

However, while studies on the relation between vowels’ formant-dispersion and the concept of dominance so far mainly looked at phonosemantic congruencies within words or texts, the focus of this study will be on sound-meaning relations between contiguous words within a given text sample. That is, we expected that words in the immediate context of target words designating smallness, delicateness, weakness, or fear would contain more high-front vowels, whereas the immediate context of target words referring to largeness, roughness, strength, or anger would contain more low and back vowels.

For clarity, we will henceforth speak of *target words* and their *contexts*, with ‘target word’ referring to a semantically categorized word that refer either to smallness or largeness and ‘context’ referring to the words that occur in the immediate proximity of the target word in a given text sample. The term ‘target word’ here only means that we used these words as search terms to extract one-line samples from a text corpus that include verbal designators of smallness, delicateness, or weakness on the one hand and largeness, roughness, and strength, on the other. We do not imply that these words are by definition of outstanding importance for the overall semantic processing of the selected text samples. We merely hypothesized that the average formant dispersion in a target word’s context should match the allocation of this target word to either side of the bipolar dominance dimension.

## Materials and methods

### Materials

We selected five attributes per semantic category as target words (Table 1). Criteria used for the selection of the attributes were: (1) They refer to one of the semantic dimensions that had previously been associated to the articulatory characteristics of vowel backness and/or height; (2) they are directly associated with one of the two poles of the dominance dimension; (3) they occur at least 30 times in the text corpus used. The latter requirement was implemented to secure a minimum of text samples per target-word. For the selection of the target words we used the online version of the *Duden*, a standard German dictionary which also provides a list of synonyms where applicable (www.duden.de/).

**Table 1:** Number of extracted text samples per target word.

SMALL			LARGE		
German	English	Samples	German	English	Samples
klein	small	182	groß	large	190
winzig / zierlich	tiny / petite	31	riesig	gigantic	52
zart	delicate	69	grob / derb	rough	39
schwach	weak	56	stark	strong	86
ängstlich / fürcht-	fearful	32	wütend / wut-	angry	34

We first selected two target words per group that most obviously represented size and strength, i.e., *klein* (small) and *schwach* (weak) for group SMALL and *groß* [large] and *stark* [strong] for group LARGE. As it had been shown that the distinction between anger and fear lies along the dominance dimension, with anger being related to dominance and fear to submissiveness [104–112, 113–115], we also added *ängstlich* [fearful] for SMALL and *wütend* [angry] for LARGE. Attempts to include further target words failed mostly because eligible words occurred far less than 30 times in the corpus. However, as most target words were sound-iconically congruent in the sense of the hypothesis (i.e., words, referring to smallness and related concepts contained vowels with a high formant dispersion and words related to largeness and related concepts contained vowels with a low formant dispersion), we additionally included sound-meaning-incongruent target words, i.e. *riesig* [gigantic] for the group LARGE and *zart* [delicate] for the group SMALL, together with their respective counterparts *winzig* [tiny] and *grob* [rough]. This allowed us to control to what extent the phonetic characteristics of the target words exerted an influence on the hypothesized phonosemantic relation between target word and context. As neither *winzig* nor *grob* had a sufficient number of occurrences in the corpus (i.e., 30 or more), we drew on additional words that are semantically and phonetically similar, i.e., *zierlich* [petite] for *winzig* and *derb* [rough] for *grob*.

For reasons of convenience we henceforth refer to the two opposite groups of attributes as SMALL vs. LARGE, although they are not limited to physical properties but also refer to a general notion of low vs. high dominance and hence represent the potency dimension of the so-called EPA model [116]. According to this model, there is a general tendency to semantically categorize concepts along the three bi-polar dimensions Evaluation (peasant-unpleasant), Potency (dominant-submissive), and Activity (active-passive). Further developing this model, Mehrabian and Russel proposed to designate the three dimensions as *Pleasure*, *Dominance*, and *Arousal* [113–115]. For our purposes, we equally rely on both variants of the EPA model and hence use the terms *Potency* and *Dominance* interchangeably.

We selected text samples from the online archive *Freiburger Anthologie* (http://freiburger-anthologie.ub.uni-freiburg.de/fa/fa.pl). The anthology comprises more than 1500 poems written between 1720 and 1890. To select the text samples, we used the stems of the attribute terms in Table 1 as search terms (e.g., ‘riesig → ‘ries-‘). We also included inflected forms, derivatives, and compound words, provided that these did not alter the meaning; thus, for *klein* (small), we also included diminutives of nouns (e.g., *Glöck-lein* (little bell)). For the phonetic analysis, we treated compound words as two separate words (e.g., *riesenhoch* → *riesen* + *hoch* [gigantically high]). To ensure that neither a specific author nor a single poem would dominate the data, we randomly selected a maximum of six samples per author and a maximum of five samples per poem for each target word. Lists of all text samples, all authors, and all titles can be found in the Supplements.

The number of occurrences in the corpus varied greatly for our target words (see Table 1). In order to avoid that the results are dominated by samples of target words with a relatively high frequency of occurrence, we randomly selected 30 text samples per target word using the shuffle algorithm in the Python programming language’s Numpy package. In contrast, when testing the effect of sound-meaning congruency for each individual target word, we included all samples detected in the corpus.

Not only the absolute length of the text samples and the position of the target words varied in our study, but also the number of words surrounding the target words. For example, when target words appeared at the beginning or at the end of a sample, their context was limited to only one side of their textual position. In fact, a majority of the samples had only one word before or after the target word, and fewer than 10% of the samples had more than two words before and after the target word (S1 Fig). Therefore, to eliminate the distance between a context word and a target word as a confounding variable, we included no more than two words before and two words after the target words in our analysis.

### Phonetic analysis of the context words

For the phonetic transcription, we used a web-based tool for grapheme-to-phoneme conversion. The tool was developed at the *Bavarian Archive of Speech Signals* in the context of the CLARIN-D project [117, 118] (https://clarin.phonetik.uni-muenchen.de/BASWebServices/interface). A manual inspection of the transcriptions suggested that the results were acceptable for the purpose of this study.

Formant dispersion was operationalized as the distance between the first two formants (dF = |F1-F2|; Table 2). To this end, Hertz frequencies for the formants were adopted from Kohler [119]. For the analysis, we converted Hertz to Mel, a psychoacoustic scale that assesses the perceptual equivalent of the physically measurable frequency [120, 121]. We used the formula introduced by O’Shaughnessy [122] for the conversion (i.e., *m = 2595 log (1 + f/700)*, with *m* = value in Mel and *f* = frequency in Hertz). Thus, all values for formant frequency in the results section are reported in Mel. Diphthongs were categorized according to their latter vowel; in this regard, we followed Greenberg and Jenkins [30], who assessed the association between vowels and diphthongs with nonacoustic attributes. Schwa (/ə/) and the near-open central vowel /ɐ/ (German: “a-Schwa” or “low-Schwa”) were not included in the analysis. We assessed the average values of the articulatory and acoustic characteristics per line for all vowels of all context words, excluding the target word.

**Table 2:** Formant frequencies for vowels in Mel.

Vowel		Frequency in Mel		
IPA	SAMPA	F1	F2	dF
i:	i:	344	1695	1351
I	I	457	1602	1145
e:	e:	530	1640	1110
ɛ	E	607	1479	871
y:	y:	344	1341	996
ʏ	Y	509	1238	729
ø:	2:	559	1291	731
oe	9	626	1291	664
u:	u:	344	740	396
ʊ	U	509	896	386
o:	o:	530	781	251
ɔ	O	626	1000	374
a:	a:	781	1125	344
a	a	781	1183	402

Note: F1: first formant, F2: second formant, dF: formant dispersion

### Data analysis

All statistical analyses were performed using R software version 3.4.3 [123]. In a first step, we compared the average frequencies of the first two formants (F1 and F2) for the semantic categories (SMALL vs. LARGE) in the reduced dataset of 30 samples per target word, using a multivariate analysis of variance (MANOVA). Approximations of F-values were calculated using the Pillai-Bartlett trace. We also conducted separate post hoc tests to compare the averaged frequencies of the first two formants and of the formant dispersion in the text samples for the two semantic categories. As we found the difference between the semantic categories to be most pronounced for formant dispersion, we subsequently conducted a logistic regression to test the predictive power of formant dispersion for the categorization of the target words as either SMALL or LARGE. To this end, we assessed the percentage of correctly categorized text samples per target word. We then conducted an additional logistic regression in which the target words were distinguished dependent on their phonetic characteristics (i.e., articulatory-acoustic properties of their central vowel) rather than dependent on their semantic categorization (i.e., SMALL vs. LARGE). Whereas a majority of six target words showed a phonosemantic congruence of phonetic characteristics and semantic category (i.e., *klein*, *winzig*, *ängstlich*, *groß*, *stark*, *grob*), two target words of the category SMALL (schwach, zart) and two target words of the category LARGE (i.e., riesig, wütend) did not show the phonetic properties expectable under the hypothesis of phonosemantic congruence. The two separate logistic regressions were aimed at comparing the relative influence of the semantic and phonetic features of the target words on the phonetic features of the content.

For the reduced dataset (30 samples per target word), we tested whether the data confirmed the assumption of homogeneity of variances and normality of distribution using the R software car package [124] and mvnormtest package [125]. We confirmed the homogeneity of the variances using Levene’s test (F1: F[1,298] = 0.957, p > .1; F2: F[1,298] < 0.311, p > .1; dF: F[1,298] = 1.065, p > .1). Additionally, we tested the homogeneity in the covariance matrix. Results show that the ratios of variances and covariances for the semantic categories (SMALL vs. LARGE) were within an acceptable range, given the relatively large sample size (S1 Table).

Inspecting the standardized skewness, we did find indications suggesting that the distribution of the data significantly deviated from normality (S2 Table). As it has been reported that departures from normality have only marginal effects on Type 1 error rates [126], we decided to stick to the parametric MANOVA. Additionally, all results were verified through robust statistics on the ranked data using the mulrank() function in the WRS package [127, 128]. In each case, the results of the nonparametric tests fully corroborated the results of the parametric tests.

## Results

We first compared the mean values of the first two formants for the two semantic categories. As expected, the averaged frequency for the first formant (F1) was higher for text samples in the category LARGE than for text samples in the category SMALL; the opposite held for the second formant (F2). Consequently, the distance between the first and second formant (dF = |F1-F2|) was on average wider for samples in the category SMALL than for those in the category LARGE (Table 3).

**Table 3:** Averaged frequencies and t-values for F1, F2, and dF.

	SMALL				LARGE				
	*N*	Mean	*SE*	CI	*N*	Mean	*SE*	CI	*t*-value
**F1**	150	518	5.4	10.7	150	539	6.2	12.2	-2.552*
**F2**	150	1370	13.7	27.0	150	1318	14.6	28.8	2.650**
**dF**	150	852	16.1	31.9	150	778	16.7	32.9	3.111**

Note. N: sample size, SE: standard error, CI: confidence interval,

* *p* < .05.

** *p* < .01.

**Fig 2:**
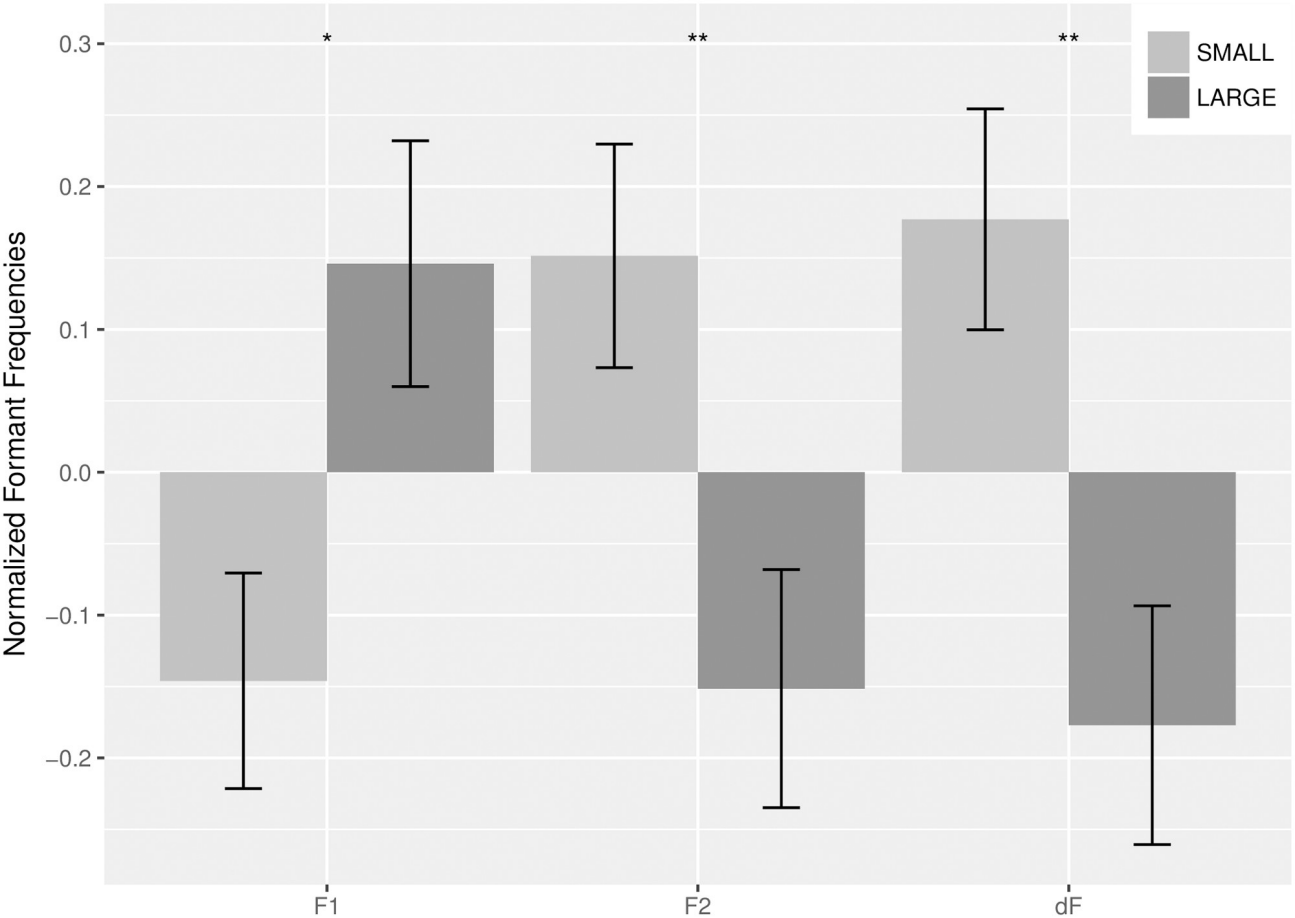
Comparison of formant frequencies for the semantic categories. Height of bars indicates normalized value of the frequency of the formant. Orientation of bars reflects their relation to the general mean for the formant. Error bars indicate standard error of mean.

For better visualization of the relation between the semantic category and the formant frequency, we compared the normalized values for F1, F2, and dF for the samples from the group SMALL and the group LARGE (Fig 2).

Testing the influence of the target words on the phonetic characteristics of their context, we found a highly significant effect of the semantic category on the frequency of the first two formants (V = 0.03, *F*[2,297] = 5.2, *p* < .01). To address the skewness of the distribution of the data, we also conducted a robust MANOVA on the ranked data [129, 130]. Results confirmed those of the parametric test (*F* = 5.98, *p* < .01, ratio of ranks [SMALL/LARGE] for F1: 0.86 and for F2: 1.19). (For results of the post hoc analysis applying the nonparametric Wilcoxon’s signed rank test, see S3 Table).

**Fig 3:**
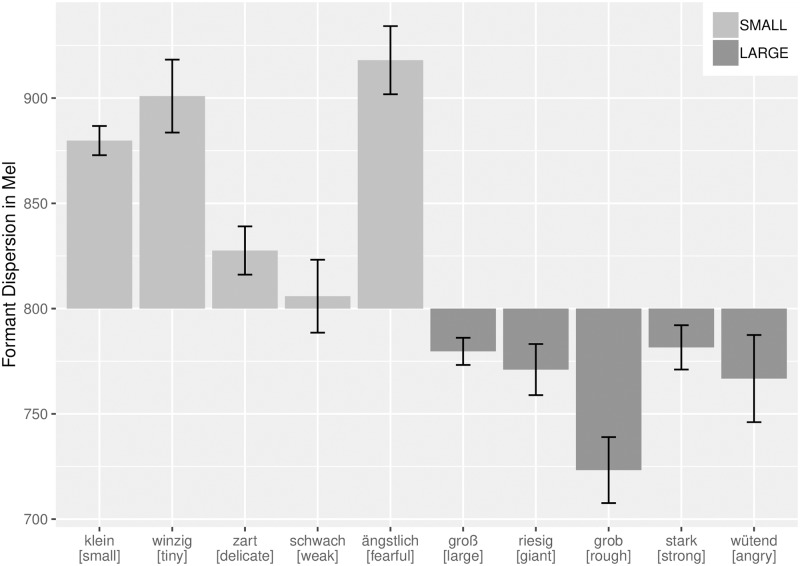
Averaged formant dispersion for target words. Error bars indicate standard error of mean.

Confirming our hypothesis, the results indicate that formant dispersion is the most reliable predictor for semantic category. To test whether this held for all target words, we compared the mean values for the formant dispersion in all text samples for each target word. Whereas we used a reduced dataset with 30 samples per target word for the MANOVA, in what follows we report the results obtained for the complete dataset including all lines found in the corpus. Again, we found a relation between the semantic category of the target word and the averaged formant dispersion in its context: all target words that are related to smallness have an averaged formant dispersion above 800 Mel whereas the opposite holds for target words that are related to largeness (Fig 3; see S4 Table for exact mean values and standard errors for each target word).

To test the extent to which the averaged formant dispersion in a target word’s context can accurately predict the semantic category of the target word, we conducted a logistic regression with formant dispersion as the predictor and semantic category as the outcome variable. Confirming our expectation, formant dispersion proved to be a significant predictor for the semantic category of the samples (*b* = .002, *z* = 6.174, *p* < .001). The resulting model explains roughly 13% of the variance (Nagelkerke’s *R*^2^ = .132). A chi-square test comparing the fit of the model in relation to a baseline model suggests a highly significant improvement (i.e., reduction of deviance; χ^2^(1) = 41.21, *p* < .001).

We also compared these results to a second model with formant dispersion as predictor and the target words grouped by their phonetic characteristics as outcome variable. To this end, we divided the target words into two groups depending on the formant dispersion of their central vowel (high formant dispersion: *klein*, *winzig*, *ängstlich*, *riesig*, *wütend*; low formant dispersion: *groß*, *stark*, *grob*, *schwach*, *zart*). As a majority of text samples came from target words that were phono-semantically congruent (i.e., *klein*, *winzig*, *ängstlich*, *groß*, *stark*, *grob*) we expected only marginal differences between these two models. Still, compared to the first model (semantic categorization) the predictive power of the second model (phonetic categorization), albeit highly significant (b = .001, z = 4.786, p < .001, Nagelkerke’s R^2^ = .078), clearly decreased.

**Fig 4:**
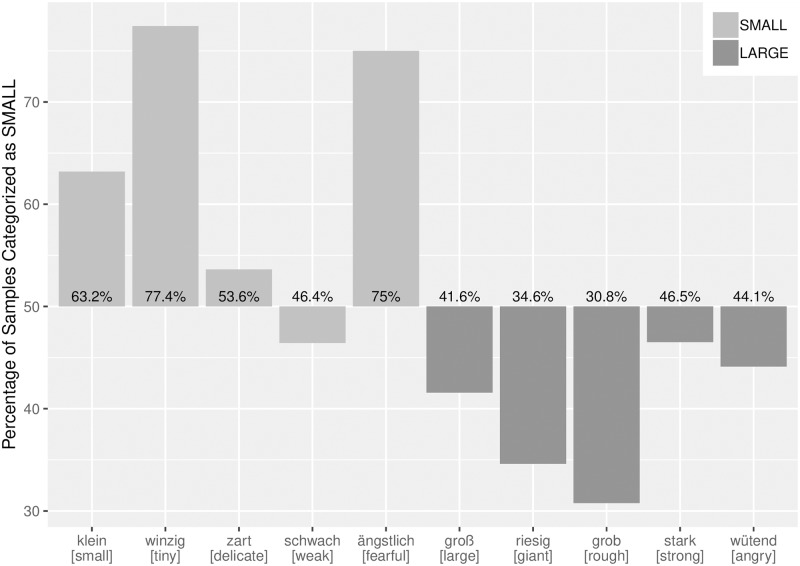
Categorization of target words. Bars represent the percentages for samples that were categorized as SMALL. Orientation of bars indicates the semantic categorization of the target word, with LARGE < 50% and SMALL > 50%.

Finally, using the averaged predicted probability of all text samples as a criterion for categorizing each sample as either SMALL or LARGE, we found that with only one exception (‘weak’), a majority of the samples for each target word were categorized in accordance with the target word’s semantic category (Fig 4). That is, the ratio of correctly vs. incorrectly categorized text samples proved to be a valid criterion for the semantic classification of the target words.

## Discussion

The results confirm our hypothesis: phonetic properties that show a sound-iconic resonance with the meaning of our target words were indeed found to be overrepresented in the words immediately preceding and/or following these target words. The averaged distance between the frequency of the first and the second formant turned out to be significantly wider in the context of attributes that designate smallness or related concepts than in the context of attributes that connote largeness or related concepts. This relation between a word’s semantic categorization with regard to the dimension of Dominance and the averaged formant dispersion in its context held for nine out of ten attributes examined. Hence the effect is fairly robust even though the concepts under consideration (e.g., *small* vs. *delicate* vs. *weak* vs. *fearful*) show substantial semantic differences. Since the abstract notion of dominance or potency, as defined in the EPA-Modell [113–115], is the uniting factor that groups these attributes into one category, our results indicate that formant dispersion is not exclusively associated with size but with a more general notion of physical and social dominance. These results are in line with previous findings that the relative distance between the first two formants is associated with either dominance or submissiveness. Thus, whether deliberately or involuntarily, acoustic properties of phonemes are apparently used in a way that emphasizes abstract notions expressed in the content of the text via contiguity-based sound iconicity.

Our results are in accord with previous findings, according to which humans tend to adapt acoustic characteristics of their voice to the meaning of an utterance [57, 58]. At the same time, we propose that contiguity-based sound iconicity, as reported here, is qualitatively different from iconic prosody or—more generally—from iconic gestures. According to Clark et al, iconic gestures are a specific kind of “performed depiction,” i.e., they visually support, or embody, the message of an utterance [131]. Adopting this approach, one could argue that the acoustic characteristics of the vowels are a physical instantiation of the concept of largeness or smallness. However, while iconic gesture and iconic prosody use distinct channels of communication, such as hand movements or pitch modulation, the acoustic characteristics of the words under scrutiny in the present study are not highlighted in any special expressive fashion. Rather, it is only by virtue of their fairly inconspicuous cooccurence, or contiguity, that a sound-iconic relation emerges between the meaning of one word and the sound of a neighboring word. Therefore, this special type of sound iconicity has escaped the attention of researchers longer than other types.

The so-called *exemplar theory* can be read to suggest a possible explanation of our results. According to this theory, the cognitive representation of concepts is structured in accordance with the perceived similarity between these concepts. Applied to language, this means that verbal tokens that are similar regarding—for example—their phonetic shape, their meaning, or their syntactic function constitute clusters which allow for comparing and categorizing new experiences [132–134]. The semantic interpretation of a stimulus as a representation of a certain concept would then follow from its positioning within a multi-dimensional feature space (in language, semantic space) and, consequently, from the semantic proximity of this new stimulus to previously stored experience. Thus, one could speculate that phonosemantic relations between the meaning of one word and the acoustic properties of another word might cause them to be memorized as tokens of a common cluster, which in turn could bias authors to use these words together. Accordingly, words which are perceived as semantically similar are indeed used in similar grammatical constructions [135].

However, to the best of our knowledge, the exemplar theory does not cover sound-meaning relations of the type investigated in our study. Research on similarity-based categorization of linguistic tokens has so far focused on similarity between different tokens of the same type, that is, similarity between phonetic features *or* similarity in meaning. In contrast, the crucial point of contiguity-based sound iconicity is precisely its emphasis on cross-fertilizations between the form and content of words on the one hand, and on the other hand across sensory modalities (e.g., sound frequency and size). Thus, at their current state, exemplar theory and contiguity-based sound iconicity do not address the same issues.

Still, while we believe that contiguity-based sound iconicity is a specific type of form-content congruency in written language, it should also be noted that the averaged formant dispersion per text sample varied greatly within each category (SMALL vs. LARGE). That is, while we did find a fairly robust contiguity-based sound-meaning resonance across all samples for the two categories, the percentage of correctly categorized text samples for three target words (i.e., ‘delicate’, ‘weak’, and ‘strong’) was only marginally above the level of chance, and, in the case of ‘weak’, even contrary to our expectation.

Thus, neighboring sound qualities do by no means always drive home the meaning of the words they surround. However, this lack of consistency in the relation between sound and meaning was to be expected for more than one reason. First, the mere occurrence of the attribute ‘giant’ in a line of poetry does not necessarily predict the message intended by the author. Given that a vowel’s formant frequency is not primarily related to size but to the notion of physical and social dominance, the text samples containing words like ‘giant’ do not necessarily suit acoustic features that imply high dominance (consider, e.g., ‘a giant failure’). In other words, to improve the predictive power of formant dispersion for semantic categorization, it might be necessary to assess the meaning of the relevant word combinations rather than basing the analysis on the mere occurrence of specific words irrespective of the context.

Second, the author of a text may make use of a wide variety of linguistic features other than contiguity-based sound iconicity that likewise allow for highlighting content in a non-semantic fashion. For example, rhyme, meter, and syntactic parallelism are all similarity-based features that poets frequently use to guide readers’ attention and also to highlight semantic relations. Thus, contiguity-based sound iconicity is but one feature for establishing form-content relations, and this specific feature might well be out of focus or even intentionally sacrificed in favor of other linguistic features, if appropriate.

Finally, it is not clear to what extent a focus on the average—such as the *averaged* formant dispersion—is a good measurement for phonosemantic relations. As Jakobson [70] pointed out, some words in a poem are more in the fore of the reader’s mind than others (p. 19). Therefore, one could speculate that rather than treating all words equally when assessing formal linguistic characteristics, the contribution of the words should be weighted according to the relative attention they receive.

The fact that most target words for SMALL and LARGE show phonosemantic properties stipulated by the hypothesis of the sound iconicity of magnitude implies a limitation for the interpretation of our results: we cannot rule out that the spreading of the respective formant dispersion to neighboring words is not only driven by the meaning of the target words, but also by their phonetic properties. It is, for example, striking that the two target words *zart* [delicate] and *schwach* [weak] which are both phonosemantically incongruent also show a relatively weak sound-meaning relation with their neighboring words.

While the importance of phonological parallelism in poetry is unquestionable, it is, however, not clear that it is specifically found in cases such as those that are the object of the present study. As for one, the predictive power of the context’s formant dispersion was clearly higher for the semantic categorization of the target words than for their categorization according to their phonetic characteristics. Moreover, Roman Jakobson [70] assumed that sound-meaning relations between a word and its immediate verbal context can also be functional for compensating mismatches between the phonetic characteristic of a word and its meaning (p. 373). The German word *riesig* [giant], for example, has /i:/ as the central vowel, which, according to our theory, would connote smallness rather than largeness. Following Jakobson, one would therefore expect a particularly high frequency of back and low vowels (i.e., vowels with a small formant dispersion) in the words surrounding *riesig*. In accord with Jakobson’s assumption, we indeed found that the predictive accuracy of formant dispersion for samples containing the attribute *groß* [large] is considerably lower than that for samples containing *riesig* [giant]. Still, further research is needed to test the extent to which sound-iconic resonances are also found in the context of target words that by themselves do not show any congruence of sound and meaning.

Our results also raise questions regarding the functions of contiguity-based sound iconicity. We propose that a sound-iconic feedback between neighboring words might render the conceptual meaning more concrete and more salient. In animal communication, acoustic characteristics that signal dominance or submissiveness also have a strong emotional component as they are used to intimidate potential opponents, or to appease dominant rivals. In a similar vein, sound iconicity in human language use can be expected to implicitly set an emotional tone that is intended to guide, or prime, the recipient’s understanding of a message. In fact, results from a recent EEG study suggest that the phonetic characteristics of a word can, relatively early in the cognitive processing of words, trigger an automatic shift of the reader’s attention towards emotionally relevant content [136]. Sound iconicity might thus provide speakers and authors with means to simultaneously express meaning at various communicative levels that, at least potentially, complement and reinforce each other.

In sum, our results provide good empirical evidence for an interrelation between form and meaning in poetic language. More precisely, we found a statistically significant relation between the meaning of a word and the phonetic characteristics of its neighboring words. In light of Roman Jakobson’s proposal that relations of contiguity constitute what rhetoric and poetics used to call metonymies [137], the type of sound iconicity we here propose might well be called *metonymic sound iconicity*.

## Outlook

The phenomenon we propose is clearly in need of additional empirical evidence. First and foremost, it needs to be tested to what extent our findings based on single lines extracted from poems extend to other types of language use. Moreover, it would be interesting to investigate whether other phonosemantic relations, such as the bouba-kiki effect [28, 31, 138–140] or the oft-claimed association between perceived pitch and brightness [35, 102, 141–143] likewise come in metonymic variants in which the iconic relation is not found within a word, but is displaced onto a relation between two neighboring words.

The effect sound iconicity exerts on readers’ cognitive processes should also be further investigated. Based on the assumption that analogies between form and content can foster the embodiment of abstract concepts, one would expect that text samples exhibiting sound iconicity have a higher potential to elicit appropriate physical responses to a given content.

Finally, the question arises whether or not sound iconicity is invariably about congruencies between sound and meaning. Could sound iconicity, for example, also be instrumental for creating meaningful oppositions between sound and meaning such that contradictory feelings are amalgamated within a single expression?

## Supporting information

S1 Fig Percentage of samples per length of context. First two bars give the percentage of samples that had the indicated number of words before or after the target word. Third bar gives the percentage of samples that had the indicated number of words before or after the target word and the same number or more words in the other position.
(TIF)

S1 Table Variance and covariance matrix with ratios. (DOCX)

S2 Table Saphiro-Wilk test for normality with skewness and kurtosis. (DOCX)

S3 Table Wilcoxon signed rank test comparing F1, F2, and dF between the semantic categories SMALL and LARGE. (DOCX)

S4 Table Formant frequencies per target word. (DOCX)

S1 Data Tab-delimited text files with data. The folder contains three files. Data used in the analysis are listed in *allSamples*.*tab*, which contains a table with one line per sample and nine columns: SampleNr (running index per sample), TitleNr (running index per title), AuthorNr (running index per author), Category (SMALL vs. LARGE), KW (the target word in English), F1 (frequency of the first formant in Mel), F2 (frequency of the second formant in Mel), dF (distance between F1 and F2), and Sample (line extracted from poem). The files *allTitles*.*tab* and *allAuthors*.*tab* list the titles and authors, respectively.
(ZIP)

## References

[1] Saussure Fd. Course in general linguistics. New York: Philosophical Library; 1959.

[2] Lockwood G, Dingemanse M. Iconicity in the lab: a review of behavioral, developmental, and neuroimaging research into sound-symbolism. Front Psychol. 2015;6: 1246 10.3389/fpsyg.2015.01246 26379581 PMC4547014

[3] Nuckolls JB. The case for sound symbolism. Annual Review of Anthropology. 1999;28(1): 225–52. 10.1146/annurev.anthro.28.1.225

[4] Perniss P, Thompson RL, Vigliocco G. Iconicity as a general property of language: evidence from spoken and signed languages. Front Psychol. 2010;1: 227 10.3389/fpsyg.2010.00227 21833282 PMC3153832

[5] Schmidtke DS, Conrad M, Jacobs AM. Phonological iconicity. Front Psychol. 2014;5: 80 10.3389/fpsyg.2014.00080 24575062 PMC3921575

[6] Svantesson JO. Sound symbolism: the role of word sound in meaning. Wiley Interdiscip Rev Cogn Sci. 2017;8(5). 10.1002/wcs.1441 28328102

[7] Aryani A, Conrad M, Schmidtke D, Jacobs A. Why ‘piss’ is ruder than ‘pee’? The role of sound in affective meaning making. PLoS One. 2018;13(6): e0198430 10.1371/journal.pone.0198430 29874293 PMC5991420

[8] Blasi DE, Wichmann S, Hammarstrom H, Stadler PF, Christiansen MH. Sound-meaning association biases evidenced across thousands of languages. Proc Natl Acad Sci USA. 2016;113(39): 10818–23. 10.1073/pnas.1605782113 27621455 PMC5047153

[9] Haynie H, Bowern C, Lapalombara H. Sound symbolism in the languages of Australia. PLoS One. 2014;9(4): e92852 10.1371/journal.pone.0092852 24752356 PMC3994004

[10] Heise DR. Sound-Meaning correlations among 1,000 English words. Language and Speech. 1966;9(1): 14–27. 10.1177/002383096600900102

[11] Körtvélyessy L. A Cross-Linguistic research into phonetic iconicity. Lexis. 2011;6 10.4000/lexis.409

[12] Ultan R. Size-sound symbolism In: Greenberg JH, editor. Universals of Human Language. 2 Stanford, CA: Stanford University Press; 1978 pp. 527–68.

[13] Westermann DH. Laut und Sinn in einigen westafrikanischen Sprachen. Archiv Für Vergleichende Phonetik. 1937;1: 154–72.

[14] Whissell C. Phonosymbolism and the emotional nature of sounds: evidence of the preferential use of particular phonemes in texts of differing emotional tone. Percept Mot Skills. 1999;89(1): 19–48. 10.2466/pms.1999.89.1.19 10544399

[15] Aryani A, Jacobs AM, Conrad M. Extracting salient sublexical units from written texts: "Emophon," a corpus-based approach to phonological iconicity. Front Psychol. 2013;4: 654 10.3389/fpsyg.2013.00654 24101907 PMC3787248

[16] Auracher J, Albers S, Zhai YH, Gareeva G, Stavniychuk T. P is for happiness, N is for sadness: Universals in sound iconicity to detect emotions in poetry. Discourse Process. 2011;48(1):1–25. 10.1080/01638531003674894

[17] Bailey RW. Statistics and the sounds of poetry. Poetics. 1971;1(1): 16–37. 10.1016/0304-422x(71)90003-9

[18] Fónagy I. Communication in poetry. Word. 1961;17(2): 194–218. 10.1080/00437956.1961.11659754

[19] Kraxenberger M, Menninghaus W. Mimological Reveries? Disconfirming the hypothesis of phono-emotional iconicity in poetry. Front Psychol. 2016;7: 1779 10.3389/fpsyg.2016.01779 27895614 PMC5109934

[20] Miall DS. Sounds of contrast: an empirical approach to phonemic iconicity. Poetics. 2001;29(1): 55–70. 10.1016/s0304-422x(00)00025-5

[21] Whissell C. Phonoemotional profiling: a description of the emotional flavour of English texts on the basis of the phonemes employed in them. Percept Mot Skills. 2000;91(2): 617–48. 10.2466/pms.2000.91.2.617 11065326

[22] Hinton L, Nichols J, Ohala JJ. Introduction: Sound-symbolism processes In: Hinton L, Nichols J, Ohala JJ, editors. Sound Symbolism. Cambridge, UK: Cambridge University Press; 1994 p. 1–12.

[23] Reay IE. Sound symbolism In: Asher RE, Simpson JMY, editors. The Encyclopedia of Language and Linguistics. 8 Oxford, England: Pergamon Press; 1994 p. 4064–70.

[24] Taylor IK, Taylor MM. Another look at phonetic symbolism. Psychological Bulletin. 1965;64(6): 413–27. 10.1037/h0022737 4159231

[25] Wescott RW. Linguistic Iconism. Language. 1971;47(2): 416–428. 10.2307/412089

[26] Auracher J. Sound iconicity of abstract concepts: Place of articulation is implicitly associated with abstract concepts of size and social dominance. PLoS One. 2017;12(11): e0187196 10.1371/journal.pone.0187196 29091943 PMC5665516

[27] Birch D, Erickson M. Phonetic symbolism with respect to three dimensions from the semantic differential. J Gen Psychol. 1958;58(2): 291–7. 10.1080/00221309.1958.9920401 13539363

[28] Bremner AJ, Caparos S, Davidoff J, de Fockert J, Linnell KJ, Spence C. "Bouba" and "Kiki" in Namibia? A remote culture make similar shape-sound matches, but different shape-taste matches to Westerners. Cognition. 2013;126(2): 165–72. 10.1016/j.cognition.2012.09.007 23121711

[29] Brown RW, Black AH, Horowitz AE. Phonetic symbolism in natural languages. The Journal of Abnormal and Social Psychology. 1955;50(3): 388–93. 10.1037/h0046820 14381156

[30] Greenberg JH, Jenkins JJ. Studies in the psychological correlates of the sound system of American English, III and IV. Word. 1966;22(1–3): 207–42. 10.1080/00437956.1966.11435451

[31] Köhler W. Gestalt psychology. New York, NY: Liveright; 1929.

[32] Kovic V, Plunkett K, Westermann G. The shape of words in the brain. Cognition. 2010;114(1): 19–28. 10.1016/j.cognition.2009.08.016 19828141

[33] Maurer D, Pathman T, Mondloch CJ. The shape of boubas: sound-shape correspondences in toddlers and adults. Dev Sci. 2006;9(3): 316–22. 10.1111/j.1467-7687.2006.00495.x 16669803

[34] Miron MS. A crosslinguistic investigation of phonetic symbolism. The Journal of Abnormal and Social Psychology. 1961;62(3): 623–30. 10.1037/h0045212 14474450

[35] Newman SS. Further experiments in phonetic symbolism. The American Journal of Psychology. 1933;45(1): 53–75. 10.2307/1414186

[36] Ozturk O, Krehm M, Vouloumanos A. Sound symbolism in infancy: evidence for sound-shape cross-modal correspondences in 4-month-olds. J Exp Child Psychol. 2013;114(2): 173–86. 10.1016/j.jecp.2012.05.004 22960203

[37] Peña M, Mehler J, Nespor M. The role of audiovisual processing in early conceptual development. Psychol Sci. 2011;22(11): 1419–21. 10.1177/0956797611421791 21960249

[38] Rummer R, Schweppe J, Schlegelmilch R, Grice M. Mood is linked to vowel type: the role of articulatory movements. Emotion. 2014;14(2): 246–50. 10.1037/a0035752 24708505

[39] Sapir E. A study in phonetic symbolism. Journal of Experimental Psychology. 1929;12(3): 225–39.

[40] Shrum LJ, Lowrey TM, Luna D, Lerman DB, Liu M. Sound symbolism effects across languages: Implications for global brand names. International Journal of Research in Marketing. 2012;29(3): 275–9. 10.1016/j.ijresmar.2012.03.002

[41] Thompson PD, Estes Z. Sound symbolic naming of novel objects is a graded function. Q J Exp Psychol (Hove). 2011;64(12): 2392–404. 10.1080/17470218.2011.605898 21895561

[42] Topolinski S, Boecker L, Erle TM, Bakhtiari G, Pecher D. Matching between oral inward-outward movements of object names and oral movements associated with denoted objects. Cogn Emot. 2017;31(1): 3–18. 10.1080/02699931.2015.1073692 26284430

[43] Usnadze D. Ein experimenteller Beitrag zum Problem der psychologischen Grundlagen der Namengebung. Psychologische Forschung. 1924;5(1): 24–43. 10.1007/bf00402395

[44] Westbury C, Hollis G, Sidhu DM, Pexman PM. Weighing up the evidence for sound symbolism: Distributional properties predict cue strength. Journal of Memory and Language. 2018;99: 122–50. 10.1016/j.jml.2017.09.006

[45] Westbury C. Implicit sound symbolism effect in lexical access, revisited: A requiem for the interference task paradigm. Journal of Articles in Support of the Null Hypothesis. 2018; 15(1): 1–12.

[46] Imai M, Kita S. The sound symbolism bootstrapping hypothesis for language acquisition and language evolution. Philos Trans R Soc Lond B Biol Sci. 2014;369(1651): 20130298 10.1098/rstb.2013.0298 25092666 PMC4123677

[47] Imai M, Kita S, Nagumo M, Okada H. Sound symbolism facilitates early verb learning. Cognition. 2008;109(1): 54–65. 10.1016/j.cognition.2008.07.015 18835600

[48] Monaghan P, Shillcock RC, Christiansen MH, Kirby S. How arbitrary is language? Philos Trans R Soc Lond B Biol Sci. 2014;369(1651): 20130299 10.1098/rstb.2013.0299 25092667 PMC4123678

[49] Perry LK, Perlman M, Lupyan G. Iconicity in English and Spanish and its relation to lexical category and age of acquisition. PLoS One. 2015;10(9): e0137147 10.1371/journal.pone.0137147 26340349 PMC4560417

[50] Kantartzis K, Imai M, Kita S. Japanese sound-symbolism facilitates word learning in English-speaking children. Cognitive Science. 2011;35(3): 575–86. 10.1111/j.1551-6709.2010.01169.x

[51] Lockwood G, Dingemanse M, Hagoort P. Sound-symbolism boosts novel word learning. J Exp Psychol Learn Mem Cogn. 2016;42(8): 1274–81. 10.1037/xlm0000235 26844577

[52] Nygaard LC, Cook AE, Namy LL. Sound to meaning correspondences facilitate word learning. Cognition. 2009;112(1): 181–6. 10.1016/j.cognition.2009.04.001 19447384

[53] Parault SJ, Schwanenflugel PJ. Sound-symbolism: a piece in the puzzle of word learning. J Psycholinguist Res. 2006;35(4): 329–51. 10.1007/s10936-006-9018-7 16752085

[54] Wrembel M. Sound Symbolism in foreign language phonological acquisition. Research in Language. 2010;8(1): 1–14. 10.2478/v10015-010-0013-6

[55] Klink RR. Creating brand names with meaning: The use of sound symbolism. Marketing Letters. 2000;11(1): 5–20. 10.1023/a:1008184423824

[56] Shrum LJ, Lowrey TM. Sounds convey meaning: The implications of phonetic symbolism for brand name construction In: Lowrey TM, editor. Psycholinguistic Phenomena in Marketing Communications. Mahwah, NJ: Lawrence Erlbaum; 2007 p. 39–58.

[57] Nygaard LC, Herold DS, Namy LL. The semantics of prosody: acoustic and perceptual evidence of prosodic correlates to word meaning. Cogn Sci. 2009;33(1): 127–46. 10.1111/j.1551-6709.2008.01007.x 21585466

[58] Perlman M, Clark N, Johansson Falck M. Iconic prosody in story reading. Cogn Sci. 2015;39(6): 1348–68. 10.1111/cogs.12190 25351919

[59] Shintel H, Nusbaum HC, Okrent A. Analog acoustic expression in speech communication. Journal of Memory and Language. 2006;55(2): 167–77. 10.1016/j.jml.2006.03.002

[60] Whissell C. Sound symbolism in Shakespeare’s sonnets: Evidence of dramatic tension in the interplay of harsh and gentle sounds. English Language and Literature Studies. 2017;7(4): 1–10. 10.5539/ells.v7n4p1

[61] Whissell C. Emotional sound symbolism and the Volta in Shakespearean and Petrarchan sonnets. English Language and Literature Studies. 2018;8(1): 1–10. 10.5539/ells.v8n1p1

[62] Arnheim R. New essays on the psychology of art. Berkeley, CA: University of California Press; 1986.

[63] Baumgarten AG. Ästhetik. Hamburg, Germany: Felix Meiner Verlag; 2007 [org. 1750].

[64] Eldridge R. Form and content: An aesthetic theory of art. The British Journal of Aesthetics. 1985;25(4): 303–16. 10.1093/bjaesthetics/25.4.303

[65] Hegel GWF. Aesthetics: Lectures on fine art. New York, NY: Oxford University Press; 1975 [org. 1820/21].

[66] Kandinsky W. Über die Formfrage In: Kandinsky W, Marc F, editors. Der Blaue Reiter. München, Germany: Piper; 1914 p. 72–102.

[67] McGregor R. Literary Thickness. The British Journal of Aesthetics. 2015;55(3): 343–60. 10.1093/aesthj/ayv019

[68] Morris CW. Esthetics and the theory of signs. Erkenntnis. 1939;8(1): 131–50. 10.1007/bf00176021

[69] Tolstoy L, Maude A. What is art? Rev. ed New York: Barnes & Noble Books; 2005 [org. 1897].

[70] Jakobson R. Closing statement: Linguistics and poetics In: Sebeok TA, editor. Style in Language. New York: Wiley; 1960 p. 350–77.

[71] Johnson RC. Magnitude symbolism of English words. Journal of Verbal Learning and Verbal Behavior. 1967;6(4): 508–11. 10.1016/s0022-5371(67)80008-2

[72] Ohtake Y, Haryu E. Investigation of the process underpinning vowel-size correspondence. Japanese Psychological Research. 2013;55(4): 390–9. 10.1111/jpr.12029

[73] Pitcher BJ, Mesoudi A, McElligott AG. Sex-biased sound symbolism in English language first names. PLoS One. 2013;8(6): e64825 10.1371/journal.pone.0064825 23755148 PMC3673912

[74] Preziosi MA, Coane JH. Remembering that big things sound big: Sound symbolism and associative memory. Cogn Res Princ Implic. 2017;2(1): 10 10.1186/s41235-016-0047-y 28275703 PMC5318481

[75] Becker JA, Fisher SK. Comparison of associations to vowel speech sounds by English and Spanish speakers. Am J Psychol. 1988;101(1): 51–7. 10.2307/1422792 2452578

[76] Shinohara K, Kawahara S. A cross-linguistic study of sound symbolism: The images of size. 36th Annual Meeting of the Berkeley Linguistic Society; Berkeley, CA. 2016. p. 396–410.

[77] Bauer L. No phonetic iconicity in evaluative morphology. Studia Linguistica. 1996;50(2): 189–206. 10.1111/j.1467-9582.1996.tb00349.x

[78] Diffloth G. i: big, a: small In: Hinton L, Nichols J, Ohala JJ, editors. Sound Symbolism. Cambridge, UK: Cambridge University Press; 1994 p. 107–14.

[79] Tsur R. Size–sound symbolism revisited. Journal of Pragmatics. 2006;38(6): 905–24. 10.1016/j.pragma.2005.12.002

[80] Ladefoged P, Disner SF. Vowels and consonants. 3 ed Chicester, UK: Wiley; 2012.

[81] Ohala JJ. An ethological perspective on common cross-language utilization of F0 of voice. Phonetica. 1984;41(1): 1–16. 10.1159/000261706 6204347

[82] Ohala JJ. The frequency code underlies the sound-symbolic use of voice pitch In: Hinton L, Nichols J, Ohala JJ, editors. Sound symbolism. Cambridge, UK: Cambridge University Press; 1994 p. 325–47.

[83] Morton ES. On the occurrence and significance of motivation-structural rules in some bird and mammal sounds. The American Naturalist. 1977;111(981): 855–69. 10.1086/283219

[84] Fitch WT. Vocal tract length and formant frequency dispersion correlate with body size in rhesus macaques. The Journal of the Acoustical Society of America. 1997;102(2): 1213–22. 10.1121/1.421048 9265764

[85] Charlton BD, Ellis WA, McKinnon AJ, Cowin GJ, Brumm J, Nilsson K, et al Cues to body size in the formant spacing of male koala (Phascolarctos cinereus) bellows: honesty in an exaggerated trait. J Exp Biol. 2011;214(Pt 20): 3414–22. 10.1242/jeb.061358 21957105

[86] Ey E, Pfefferle D, Fischer J. Do age- and sex-related variations reliably reflect body size in non-human primate vocalizations? A review. Primates. 2007;48(4): 253–67. 10.1007/s10329-006-0033-y 17226064

[87] Jones MR, Witt CC. Utility of vocal formant spacing for monitoring sandhill crane subspecies. Wildlife Society Bulletin. 2012;36(1): 47–53. 10.1002/wsb.110

[88] Riede T, Fitch T. Vocal tract length and acoustics of vocalization in the domestic dog (Canis familiaris). Journal of Experimental Biology. 1999;202(20):2859–67. 10504322 10.1242/jeb.202.20.2859

[89] Sanvito S, Galimberti F, Miller EH. Vocal signalling of male southern elephant seals is honest but imprecise. Anim Behav. 2007;73(2): 287–99. 10.1016/j.anbehav.2006.08.005

[90] Vannoni E, McElligott AG. Low frequency groans indicate larger and more dominant fallow deer (Dama dama) males. PLoS One. 2008;3(9): e3113 10.1371/journal.pone.0003113 18769619 PMC2518835

[91] Watkins CD, Pisanski K. Vocal indicators of dominance. 2016:1–6.

[92] Evans S, Neave N, Wakelin D. Relationships between vocal characteristics and body size and shape in human males: an evolutionary explanation for a deep male voice. Biol Psychol. 2006;72(2): 160–3. 10.1016/j.biopsycho.2005.09.003 16280195

[93] Fitch WT, Giedd J. Morphology and development of the human vocal tract: A study using magnetic resonance imaging. The Journal of the Acoustical Society of America. 1999;106(3): 1511–22. 10.1121/1.427148 10489707

[94] González J. Formant frequencies and body size of speaker: a weak relationship in adult humans. Journal of Phonetics. 2004;32(2): 277–87. 10.1016/s0095-4470(03)00049-4

[95] Charlton BD, Reby D. The evolution of acoustic size exaggeration in terrestrial mammals. Nat Commun. 2016;7: 12739 10.1038/ncomms12739 27598835 PMC5025854

[96] Leongomez JD, Mileva VR, Little AC, Roberts SC. Perceived differences in social status between speaker and listener affect the speaker‘s vocal characteristics. PLoS One. 2017;12(6): e0179407 10.1371/journal.pone.0179407 28614413 PMC5470693

[97] Puts DA, Hodges CR, Cárdenas RA, Gaulin SJC. Men‘s voices as dominance signals: vocal fundamental and formant frequencies influence dominance attributions among men. Evolution and Human Behavior. 2007;28(5): 340–4. 10.1016/j.evolhumbehav.2007.05.002

[98] Collins SA. Men‘s voices and women‘s choices. Anim Behav. 2000;60(6): 773–80. 10.1006/anbe.2000.1523 11124875

[99] Feinberg DR, Jones BC, DeBruine LM, O’Connor JJM, Tigue CC, Borak DJ. Integrating fundamental and formant frequencies in women’s preferences for men’s voices. Behavioral Ecology. 2011;22(6): 1320–5. 10.1093/beheco/arr134

[100] Hodges-Simeon CR, Gaulin SJ, Puts DA. Different vocal parameters predict perceptions of dominance and attractiveness. Hum Nat. 2010;21(4): 406–27. 10.1007/s12110-010-9101-5 21212816 PMC2995855

[101] Xu Y, Lee A, Wu WL, Liu X, Birkholz P. Human vocal attractiveness as signaled by body size projection. PLoS One. 2013;8(4): e62397 10.1371/journal.pone.0062397 23638065 PMC3634748

[102] Fischer-Jørgensen E. Perceptual dimensions of vowels. STUF—Language Typology and Universals. 1968;21(1–6): 94–8. 10.1524/stuf.1968.21.16.94

[103] Clark MS, Pataki SP, Carver VH. Some thoughts and findings on self-presentation of emotions in relationships In: Fletcher GJO, Fitness J, editors. Knowledge structures in close relationships: A social psychological approach. Hillsdale, NJ: Lawrence Erlbaum Associates; 1996 p. 247–74.

[104] Öhman A, Dimberg U, Öst LG. Animal and social phobias: Biological constraints on learned fear responses In: Reiss S, Bootzin RR, editors. Theoretical issues in behavior therapy. New York, NY: Academic Press; 1985 p. 123–75.

[105] Öhman A, Mineka S. Fears, phobias, and preparedness: Toward an evolved module of fear and fear learning. Psychological Review. 2001;108(3): 483–522. 11488376 10.1037/0033-295x.108.3.483

[106] Cabral JCC, Tavares PS, de Almeida RMM. Reciprocal effects between dominance and anger: A systematic review. Neurosci Biobehav Rev. 2016;71: 761–71. 10.1016/j.neubiorev.2016.10.021 27984056

[107] Carney DR, Hall JA, LeBeau LS. Beliefs about the nonverbal expression of social power. Journal of Nonverbal Behavior. 2005;29(2): 105–23. 10.1007/s10919-005-2743-z

[108] Hareli S, Shomrat N, Hess U. Emotional versus neutral expressions and perceptions of social dominance and submissiveness. Emotion. 2009;9(3): 378–84. 10.1037/a0015958 19485615

[109] Knutson B. Facial expressions of emotion influence interpersonal trait inferences. Journal of Nonverbal Behavior. 1996;20(3): 165–82. 10.1007/bf02281954

[110] Sewards TV, Sewards MA. Fear and power-dominance drive motivation: neural representations and pathways mediating sensory and mnemonic inputs, and outputs to premotor structures. Neuroscience & Biobehavioral Reviews. 2002;26(5): 553–79. 10.1016/s0149-7634(02)00020-9 12367590

[111] Tiedens LZ. Anger and advancement versus sadness and subjugation: The effect of negative emotion expressions on social status conferral. Journal of Personality and Social Psychology. 2001;80(1): 86–94. 11195894

[112] Tracy JL, Randles D, Steckler CM. The nonverbal communication of emotions. Current Opinion in Behavioral Sciences. 2015;3: 25–30. 10.1016/j.cobeha.2015.01.001

[113] Russell JA, Mehrabian A. Evidence for a three-factor theory of emotions. Journal of Research in Personality. 1977;11(3): 273–94. 10.1016/0092-6566(77)90037-x

[114] Mehrabian A, Russell JA. An approach to environmental psychology. Cambridge, MA: MIT Press; 1974.

[115] Bakker I, van der Voordt T, Vink P, de Boon J. Pleasure, Arousal, Dominance: Mehrabian and Russell revisited. Current Psychology. 2014;33(3): 405–21. 10.1007/s12144-014-9219-4

[116] Osgood CE, Suci GJ, Tannenbaum PH. The measurement of meaning. Urbana, Ill: Univ. of Illinois Press; 1957.

[117] Reichel UD. PermA and Balloon: Tools for string alignment and text processing. Interspeech, 13th Annual Conference of the International Speech Communication Association; Portland, Oregon; 2012.

[118] Reichel UD, Kisler T. Language-independent grapheme-phoneme conversion and word stress assignment as a web service In: Hoffmann R, editor. Elektronische Sprachverarbeitung. Studientexte zur Sprachkommunikation. Dresden, Germany: TU Dresden Press; 2014.

[119] Kohler KJ. Einführung in die Phonetik des Deutschen [Introduction to German phonetics]. Berlin, Germany: Schmidt Verlag; 1995.

[120] Stevens SS, Volkmann J. The relation of pitch to frequency: A revised scale. The American Journal of Psychology. 1940;53(3): 329–53. 10.2307/1417526

[121] Stevens SS, Volkmann J, Newman EB. A scale for the measurement of the psychological magnitude pitch. The Journal of the Acoustical Society of America. 1937;8(3): 185–90. 10.1121/1.1915893

[122] O'Shaughnessy D. Speech communication: Human and machine. Reading, MA: Addison-Wesley; 1987.

[123] Core Team R. R: A language and environment for statistical computing. Vienna, Austria: R foundation for statistical computing; 2017 https://www.R-project.org.

[124] Fox J, Weisberg S. An R companion to applied regression. Thousand Oaks, CA: Sage; 2011.

[125] Jarek S. Mvnormtest: Normality test for multivariate variables. 0.1–9 ed2012.

[126] Bray JH, Maxwell SE. Multivariate analysis of variance. Beverly Hills: Sage Publications; 1985.

[127] Wilcox RR. Introduction to robust estimation and hypothesis testing 3ed Amsterdam, Netherlands: Academic Press; 2012.

[128] Wilcox RR, Schönbrodt FD. The WRS package for robust statistics in R. 0.30.1 ed2017.

[129] Brunner E, Munzel U. Nicht-parametrische Datenanalyse: Unverbundene Stichproben [Nonparametric analysis of data: Independent samples]. Berlin, Germany: Springer Verlag; 2013.

[130] Munzel U, Brunner E. Nonparametric Tests in the Unbalanced Multivariate One‐Way Design. Biometrical Journal. 2000;42(7):837±54.

[131] Clark HH. Depicting as a method of communication. Psychol Rev. 2016;123(3): 324–47. 10.1037/rev0000026 26855255

[132] Bybee JL. From usage to grammar: The mind‘s response to repetition. Language. 2006;82(4): 711–33. 10.1353/lan.2006.0186

[133] Johnson K. Speech perception without speaker normalization: An exemplar model In: Johnson K, Mullennix JW, editors. Talker variability in speech processing. San Diego, CA: Academic Press; 1997 p. 145–65.

[134] Pierrehumbert JB. Exemplar dynamics: Word frequency, lenition and contrast In: Bybee JL, Hopper PJ, editors. Frequency effects and the emergence of linguistic structure. 45 Amsterdam: John Benjamins; 2001 p. 137–58.

[135] Bybee JL, Eddington D. A Usage-based approach to Spanish verbs of ‘becoming’. Language. 2006;82(2): 323–55. 10.1353/lan.2006.0081

[136] Ullrich S, Kotz SA, Schmidtke DS, Aryani A, Conrad M. Phonological iconicity electrifies: An ERP study on affective sound-to-meaning correspondences in German. Front Psychol. 2016;7: 1200 10.3389/fpsyg.2016.01200 27588008 PMC4988991

[137] Jakobson R. Two aspects of language: Metaphor and Metonymy In: Gras VW, editor. European Literary Theory and Practice: From Existential Phenomenology to Structuralism. New York, NY: Delta; 1973.

[138] Fort M, Martin A, Peperkamp S. Consonants are more important than vowels in the bouba-kiki effect. Lang Speech. 2015;58(Pt 2): 247–66. 10.1177/0023830914534951 26677645

[139] Kovic V, Plunkett K, Westermann G. The shape of words in the brain. Cognition. 2010;114(1): 19–28. 10.1016/j.cognition.2009.08.016 19828141

[140] Ramachandran VS, Hubbard EM. Synaesthesia—A window into perception, thought and language. Journal of Consciousness Studies. 2001;8(12): 3–34.

[141] Ludwig VU, Adachi I, Matsuzawa T. Visuoauditory mappings between high luminance and high pitch are shared by chimpanzees (Pan troglodytes) and humans. Proc Natl Acad Sci U S A. 2011;108(51): 20661–5. 10.1073/pnas.1112605108 22143791 PMC3251154

[142] Marks LE. On associations of light and sound: the mediation of brightness, pitch, and loudness. Am J Psychol. 1974;87(1–2): 173–88. 10.2307/1422011 4451203

[143] Tsur R. Sound affects of poetry: Critical impressionism, reductionism and cognitive poetics. Pragmatics & Cognition. 1997;5(2): 283–304. 10.1075/pc.5.2.05tsu

