# Supplementary figures and images for: Contiguity-based sound iconicity: The meaning of words resonates with phonetic properties of their immediate verbal contexts
Source: PLoS One. 2019 May 16;14(5):e0216930. doi: 10.1371/journal.pone.0216930 (PMC6522027; doi:10.1371/journal.pone.0216930)

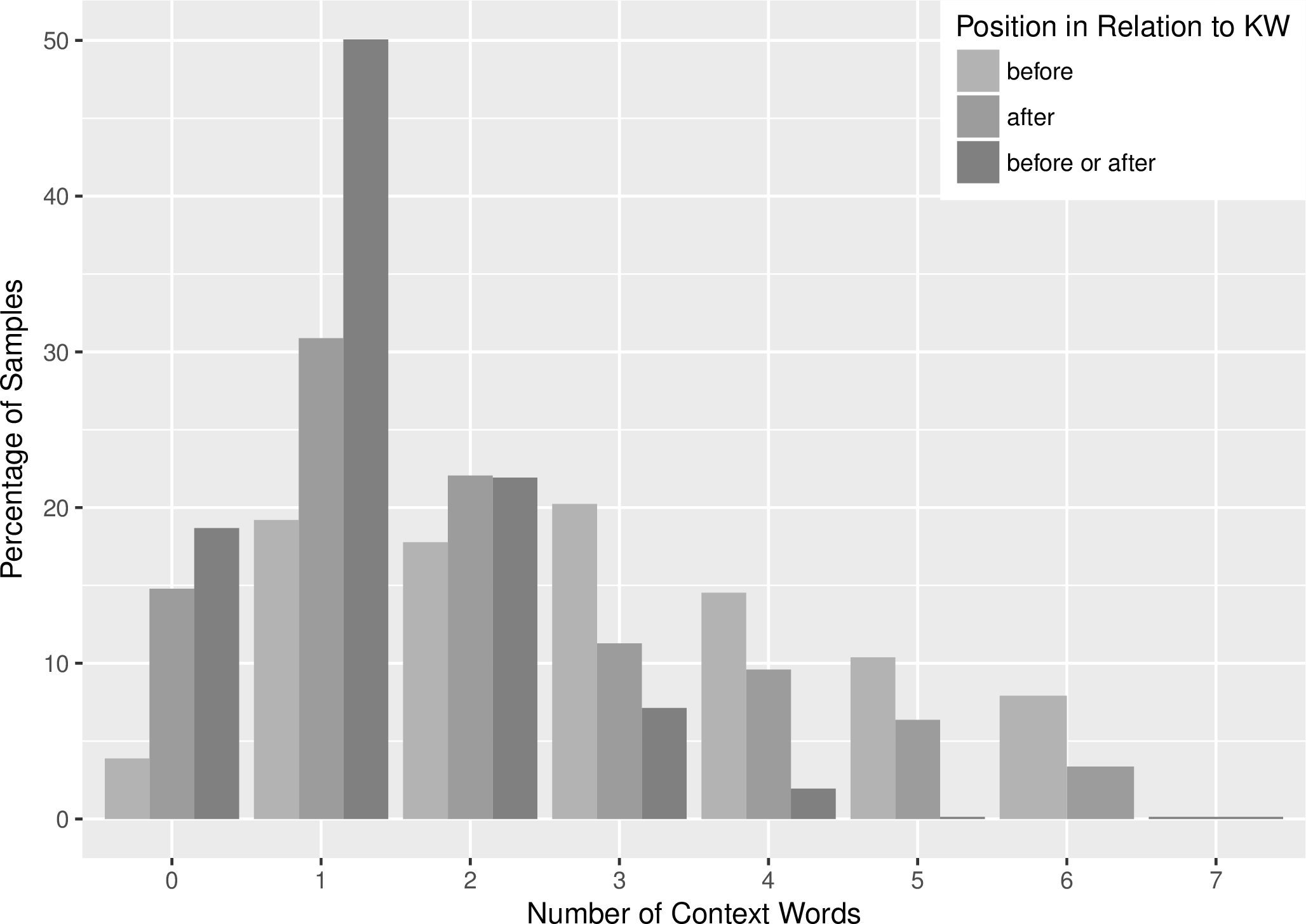

Supplement: S1 Fig — First two bars give the percentage of samples that had the indicated number of words before or after the target word. Third bar gives the percentage of samples that had the indicated number of words before or after the target word and the same number or more words in the other position. (TIF) [file pone.0216930.s001.tif]
