# Supplementary material for: Contiguity-based sound iconicity: The meaning of words resonates with phonetic properties of their immediate verbal contexts
Source: PLoS One. 2019 May 16;14(5):e0216930. doi: 10.1371/journal.pone.0216930 (PMC6522027; doi:10.1371/journal.pone.0216930)
Supplement: S1 Table — (DOCX) [file pone.0216930.s002.docx]

**S1 Table. Variance and covariance matrix with ratios.**

|  | **Variance** | | **Covariance** |
| --- | --- | --- | --- |
|  | **F1** | **F2** |  |
| **SMALL** | 4381.8 | 27971.0 | -3339.2 |
| **LARGE** | 5706.6 | 31779.9 | -4069.7 |
| **ratio** | 1.302 | 1.136 | 1.219 |
