# Supplementary material for: Contiguity-based sound iconicity: The meaning of words resonates with phonetic properties of their immediate verbal contexts
Source: PLoS One. 2019 May 16;14(5):e0216930. doi: 10.1371/journal.pone.0216930 (PMC6522027; doi:10.1371/journal.pone.0216930)
Supplement: S2 Table — (DOCX) [file pone.0216930.s003.docx]

**S2 Table. Saphiro-Wilk test for normality with skewness and kurtosis.**

|  | **SMALL** | | | **LARGE** | | |
| --- | --- | --- | --- | --- | --- | --- |
|  | **F1** | **F2** | **dF** | **F1** | **F2** | **dF** |
| **Skewness** | 0.026 | -0.523 | -0.309 | 0.144 | -0.362 | -0.110 |
| **Skewness / 2SE** | 0.068 | **-1.320** | -0.781 | 0.363 | -0.913 | --0.278 |
| **Kurtosis** | -0.642 | 0.055 | -0.228 | -0.037 | -0.136 | -0.370 |
| **Kurtosis / 2SE** | -0.988 | 0.973 | -0.289 | 0.047 | -0.173 | -0.470 |
| **Saphiro-Wilk** | 0.99^ns^ | 0.97^**^ | 0.98^+^ | 0.99^ns^ | 0.98^+^ | 0.99^ns^ |

*Note.* Values above the threshold of ±1 are indicated in bold face.

^ns^ *p* > .1. ^+^ *p* < .1. * *p* < .05. ** *p* < .01. *** *p* < .001.
