# Supplementary material for: Contiguity-based sound iconicity: The meaning of words resonates with phonetic properties of their immediate verbal contexts
Source: PLoS One. 2019 May 16;14(5):e0216930. doi: 10.1371/journal.pone.0216930 (PMC6522027; doi:10.1371/journal.pone.0216930)
Supplement: S3 Table — (DOCX) [file pone.0216930.s004.docx]

**S3 Table. Wilcoxon signed rank test comparing F1, F2, and dF between the semantic categories SMALL and LARGE.**

|  | **SMALL** | | | **LARGE** | | |  |  |  |
| --- | --- | --- | --- | --- | --- | --- | --- | --- | --- |
|  | ***Mdn*** | **25%** | **75%** | ***Mdn*** | **25%** | **75%** |  | ***W*** | ***r*** |
| **F1** | 523 | 462 | 574 | 541 | 489 | 577 |  | 9557^*^ | -0.13 |
| **F2** | 1398 | 1254 | 1480 | 1328 | 1200 | 1450 |  | 13194^**^ | -0.15 |
| **dF** | 876 | 735 | 971 | 788 | 636 | 896 |  | 13610^**^ | -0.18 |

*Note.* *Mdn*: median; 25%: first quartile; 75%: third quartile; *W*: test statistic; *r*: effect size.

* *p* < .05. ** *p* < .01. *** *p* < .001
