# Supplementary material for: Contiguity-based sound iconicity: The meaning of words resonates with phonetic properties of their immediate verbal contexts
Source: PLoS One. 2019 May 16;14(5):e0216930. doi: 10.1371/journal.pone.0216930 (PMC6522027; doi:10.1371/journal.pone.0216930)
Supplement: S4 Table — (DOCX) [file pone.0216930.s005.docx]

**S4 Table. Formant frequencies per target word**

| **Semantic Category** | **Target words** | |  | **F1** |  | **F2** |  | **dF** |  |
| --- | --- | --- | --- | --- | --- | --- | --- | --- | --- |
|  | **German** | **English** | ***N*** | ***M*** | ***SE*** | ***M*** | ***SE*** | ***M*** | ***SE*** |
| **SMALL** | **klein** | **small** | 182 | 512 | 5.5 | 1392 | 11.4 | 880 | 13.9 |
|  | **winzig** | **tiny** | 31 | 508 | 11.8 | 1409 | 27.1 | 901 | 34.6 |
|  | **zart** | **delicate** | 69 | 523 | 7.3 | 1351 | 19.1 | 828 | 22.9 |
|  | **schwach** | **weak** | 56 | 523 | 10.6 | 1330 | 29.9 | 806 | 34.6 |
|  | **ängstlich** | **fearful** | 32 | 511 | 12.2 | 1429 | 25.7 | 918 | 32.4 |
| **LARGE** | **groß** | **large** | 190 | 546 | 5.1 | 1326 | 11.5 | 780 | 12.9 |
|  | **riesig** | **giant** | 52 | 553 | 9.2 | 1324 | 20.7 | 771 | 24.3 |
|  | **grob** | **rough** | 39 | 560 | 13.4 | 1284 | 24.8 | 723 | 31.3 |
|  | **stark** | **strong** | 86 | 534 | 7.8 | 1316 | 18.2 | 782 | 20.1 |
|  | **wütend** | **angry** | 34 | 540 | 10.3 | 1307 | 36.6 | 767 | 41.4 |

*Note.* *N*: sample size; *M*: mean; *SE*: standard error.
